# Intron-loss evolution of hatching enzyme genes in Teleostei

**DOI:** 10.1186/1471-2148-10-260

**Published:** 2010-08-27

**Authors:** Mari Kawaguchi, Junya Hiroi, Masaki Miya, Mutsumi Nishida, Ichiro Iuchi, Shigeki Yasumasu

**Affiliations:** 1 Atmosphere and Ocean Research Institute, The University of Tokyo, 5-1-5 Kashiwanoha, Kashiwa, Chiba 277-8564, Japan; 2 Research Fellow of the Japan Society for the Promotion of Science (JSPS), Japan; 3 Department of Anatomy, St. Marianna University School of Medicine, 2-16-1 Sugao, Miyamae-ku, Kawasaki 216-8511, Japan; 4 Department of Zoology, Natural History Museum & Institute, Chiba, 955-2 Aoba-cho, Chuo-ku, Chiba 260-8682, Japan; 5 Department of Materials and Life Sciences, Faculty of Science and Technology, Sophia University, 7-1 Kioi-cho, Chiyoda-ku, Tokyo 102-8554, Japan

## Abstract

**Background:**

Hatching enzyme, belonging to the astacin metallo-protease family, digests egg envelope at embryo hatching. Orthologous genes of the enzyme are found in all vertebrate genomes. Recently, we found that exon-intron structures of the genes were conserved among tetrapods, while the genes of teleosts frequently lost their introns. Occurrence of such intron losses in teleostean hatching enzyme genes is an uncommon evolutionary event, as most eukaryotic genes are generally known to be interrupted by introns and the intron insertion sites are conserved from species to species. Here, we report on extensive studies of the exon-intron structures of teleostean hatching enzyme genes for insight into how and why introns were lost during evolution.

**Results:**

We investigated the evolutionary pathway of intron-losses in hatching enzyme genes of 27 species of Teleostei. Hatching enzyme genes of basal teleosts are of only one type, which conserves the 9-exon-8-intron structure of an assumed ancestor. On the other hand, otocephalans and euteleosts possess two types of hatching enzyme genes, suggesting a gene duplication event in the common ancestor of otocephalans and euteleosts. The duplicated genes were classified into two clades, clades I and II, based on phylogenetic analysis. In otocephalans and euteleosts, clade I genes developed a phylogeny-specific structure, such as an 8-exon-7-intron, 5-exon-4-intron, 4-exon-3-intron or intron-less structure. In contrast to the clade I genes, the structures of clade II genes were relatively stable in their configuration, and were similar to that of the ancestral genes. Expression analyses revealed that hatching enzyme genes were high-expression genes, when compared to that of housekeeping genes. When expression levels were compared between clade I and II genes, clade I genes tends to be expressed more highly than clade II genes.

**Conclusions:**

Hatching enzyme genes evolved to lose their introns, and the intron-loss events occurred at the specific points of teleostean phylogeny. We propose that the high-expression hatching enzyme genes frequently lost their introns during the evolution of teleosts, while the low-expression genes maintained the exon-intron structure of the ancestral gene.

## Background

Many of the nuclear genes of eukaryotes are composed of coding sequences (exons) interspersed with intervening sequences (introns) [1,2]. Both exon and intron sequences are transcribed into pre-mRNA, and the introns are removed from the pre-mRNA through splicing. The intron insertion sites in homologous genes have been considered to be well conserved among eukaryotes. However, recent large-scale comparisons of intron positions in orthologous genes have revealed that intron positioning varies more dynamically than previously supposed [3]. A considerable number of intron losses and gains during the evolution of eukaryotes are reported [4]. Nematodes have a particularly high rate of intron turnover [5]. In vertebrates, however, the exon-intron structures of orthologs are reported to be relatively stable [6,7]. In teleosts, Venkatesh et al. [8] compared exon-intron structures of eight genes, and showed that changes in intron positioning occurred at specific points of evolution. Although other studies [9,10] have been done, there remain many uncertainties in understanding the mechanisms and selective pressures that mediate evolutionary loss and gain of introns [11].

Our previous analyses implied that hatching enzyme genes lost their introns frequently during the evolution of teleosts [12]. Hatching enzyme is an enzyme that digests egg envelope at embryo hatching [13]. The enzyme belongs to the astacin metallo-protease family [14] as do BMP1 (bone morphogenetic protein 1) and meprin (metallo-endopeptidase from renal tissue). Hatching enzyme orthologs are also found in other vertebrates [15-18]. Those of tetrapods are multi-domain proteins consisting of astacin protease domain and additional C-terminal domain(s). In amphibians, reptiles and birds, there are one or two C-terminal CUB domains, whereas in mammals there is an unknown C-terminal domain. On the other hand, the hatching enzymes of teleosts are composed of only the protease domain [14,19,20]. The exon-intron structures of the genes coding the pre-pro-peptide and protease domain were conserved between tetrapods and Japanese eel [12], a basal teleost. Therefore, it is reasonable to suggest that the eel genes maintain the structure of the ancestral hatching enzyme genes. In the higher teleosts, however, the hatching enzyme genes were found to have frequently lost their introns [12]. Such frequent intron losses were found only in hatching enzyme gene orthologs, and not in their paralogous genes [21].

Teleostei is one of the diversified groups in vertebrates with over 26,000 living species [22] and is estimated to have diverged 284-333 million years ago (MYA) [23]. Recently, the phylogenetic relationships among teleosts have been extensively studied using whole mitochondrial DNA sequences [23-27]. The analyses make it possible to clarify gene evolution in teleosts tracing back to about 300 MYA. We compared the exon-intron structures of hatching enzyme genes in a wide variety of teleosts, and found that hatching enzyme genes lost their introns at specific points in teleostean phylogeny.

## Results and Discussion

To determine the evolutionary pathway of structural changes of hatching enzyme genes in Teleostei, we cloned the genes of 27 species including 15 newly-cloned species, as described in Materials and Methods. The species examined are distributed within the Teleostei, from basal to higher, as follows: 2 species in 1 order of Osteoglossomorpha, 4 species in 4 orders of Elopomorpha, 8 species in 6 orders of Otocephala, and 13 species in 11 orders of Euteleostei (Additional file 1).

### Diversification of hatching enzyme genes

**Figure 1:**
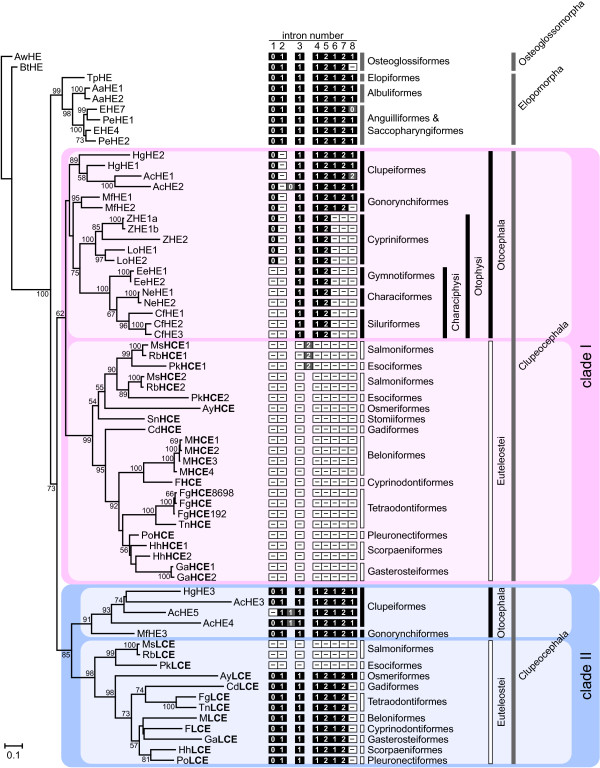
**Phylogenetic tree and exon-intron structure of hatching enzyme genes**. The ML tree was constructed from nucleotide sequences of mature enzyme portions of teleostean hatching enzyme genes using osteoglossiform gene (*AwHE*) as an outgroup. Numbers at the nodes show bootstrap support values, and the values under 50% are removed. Exon-intron structures are shown at the right of gene names. The intron numbers corresponding to the assumed ancestral hatching enzyme gene are shown at the top. The numbers in boxes represent intron phases, and the boxes are colored black for conserved introns, white for lost introns, and gray for inserted introns or introns having intron phase different from that of the ancestral gene.

A maximum likelihood tree was constructed from nucleotide sequences of the protease domains of hatching enzyme genes of all the teleosts examined (Figure 1). The tree was divided into two clades: Elopomorpha gene clade, and Otocephala and Euteleostei (Clupeocephala) gene clade. Elopomorph genes form a monophyletic clade with short branch lengths. On the other hand, the clupeocephalan gene clade was divided into two clades, named clades I and II. Within each clade, two subclades, otocephalan and euteleostean subclades, were present. These results suggest that duplication of hatching enzyme genes occurred in the ancestor of Clupeocephala (Figure 1). This duplication event is supported by reconstruction of ancestral states by the Notung program [28,29], along with further duplication events in several lineages (data not shown). Our present analysis of hatching enzyme genes shows that basal teleosts possessed a single type of gene. After the branching off of clupeocephalans from the ancestor, gene duplication occurred in the ancestor of clupeocephalans and the genes were diversified into two types.

Clade I genes were cloned from all of the clupeocephalans examined. The branching patterns of the genes within the clade (Figure 1) mirrored molecular phylogenetic relationships estimated using whole mitochondrial DNA sequences [24,25,27]. Clade II genes were also cloned from euteleosts. In otocephalans, on the other hand, the genes were cloned only from clupeiforms and gonorynchiforms, but not from otophysans (cypriniforms, characiforms, gymnotiforms and siluriforms) (Figure 1). This was corroborated by the fact that clade II genes were not found in the genome sequences of zebrafish (cypriniforms), i.e., only three clade I genes (two *ZHE1 *and one *ZHE2*) were present in the genome, and the clade II gene was absent [12]. In addition, we have recently demonstrated that the hatching of zebrafish embryos involved only ZHE1 enzymes that were expressed from the clade I genes (*ZHE1*) [30]. These results suggest that the clade II genes disappeared specifically in the otophysans lineage.

In protein level studies of euteleostean hatching enzymes, two types of hatching enzymes have been well characterized using medaka and killifish [31-34]. They have been called HCE and LCE based on how they digest egg envelope. The HCE and LCE gene orthologs were cloned from other euteleosts, and they were located in clades I and II, respectively, in the present phylogenetic tree (Figure 1).

### Intron-loss evolution of hatching enzyme genes in Teleostei

As mapped on the phylogenetic tree (Figure 1), exon-intron structures of hatching enzyme genes showed characteristic intron-loss patterns during evolution. In this section, exon-intron structures of (1) Osteoglossomorpha and Elopomorpha hatching enzyme genes, (2) Clupeocephala clade I genes and (3) Clupeocephala clade II genes will be described, and (4) their evolution will be discussed.

#### (1) Osteoglossomorpha and Elopomorpha genes

Exon-intron structures of osteoglossomorph and elopomorph genes were well conserved with only a few exceptions (*EHE7 *and *PeHE1*; Figure 1 and Additional file 2). The genes were composed of 9 exons interrupted by 8 introns (Figure 1). Exon 1 to the middle of exon 4 encode the pre-pro-peptide region, the remainder of exon 4 through to exon 8 encode the entire protease domain, and exon 9, in addition to the whole 3'-UTR, encode only two nucleotides of stop codon (*EHE4*) or 4 additional amino acids at the C-terminus of the protease domain (*EHE7*). Because of the low sequence similarity of the 9th exon, it was frequently difficult to directly determine its position in genomic DNA. However, the existence of the 8th intron was predicted by the presence of a consensus sequence of 5'-splice-site "GT" [35]. This "GT" was found in osteoglossomorph and elopomorph genes, with the exception that exon 8 of *BtHE *gene comprised coding sequence and stop codon. From these results, we concluded that the 9th exon was evidently present in the genes. These exon-intron structures, including intron phases (the positions of intron between or within codons) [36], were the same as those of tetrapod genes [12], suggesting that the ancestral teleostean hatching enzyme genes had a 9-exon-8-intron structure.

#### (2) Clupeocephala clade I genes

Among the duplicated genes of clupeocephalans, clade I and II genes, the clade I genes had variable exon-intron structures different from that of the ancestral gene. Figure 1 suggests that otocephalan clade I genes lost their introns successively during evolution. In clupeiforms and gonorynchiforms, although their exon-intron structure was similar to that of the ancestral gene, one intron (2nd intron of the ancestral genes) was commonly lost and they possessed an 8-exon-7-intron structure with two exceptions (*AcHE2 *and *MfHE2*) (Figure 1). In cypriniforms, the clade I genes possessed a 5-exon-4-intron structure: this structure appeared to be derived by the additional loss of 3 more introns (6th, 7th and 8th introns) (Figure 1), without any nucleotide deletions or insertions in exonic regions (Additional file 2). The clade I genes of characiphysans (characiforms, gymnotiforms and siluriforms) lost one more intron (1st intron), resulting a 4-exon-3-intron structure (Figure 1).

In contrast to the otocephalan genes, euteleostean clade I genes, except in salmoniforms and esociforms, had no introns, resulting in an intron-less structure (Figure 1). Salmoniforms and esociforms possess two clade I genes. One each of these (*MsHCE1*, *RbHCE1 *and *PkHCE1*) was composed of 2 exons interrupted by 1 intron, resulting a 2-exon-1-intron structure, while the other (*MsHCE2*, *RbHCE2 *and *PkHCE2*) showed the intron-less structure (Figure 1). The intron insertion sites of the former genes did not correspond to those of any other hatching enzyme genes (Additional file 2), suggesting that, after all introns were lost in the ancestor of euteleosts, duplication occurred in the common ancestor of salmoniforms and esociforms, and then, one intron was newly inserted into one of the two clade I genes.

#### (3) Clupeocephala clade II genes

Different from clade I genes, exon-intron structure of clade II genes was relatively stable. Otocephalan clade II genes were composed of 9 exons and 8 introns (the same as the ancestral gene; Figure 1), with exceptions seen in anchovy genes (*AcHE4 *and *AcHE5*), whose intron loss/gain has been demonstrated to occur specifically in the anchovy lineage [37]. Most of euteleostean clade II genes had exon-intron structures similar to the ancestral gene, although an 8th intron loss was observed. Salmoniform and esociform genes, however, were quite different from the others, showing an intron-less structure. The intron-loss might have occurred specifically in the common ancestor of salmoniforms and esociforms.

#### (4) Evolutionary pathway of change of exon-intron structure

**Figure 2:**
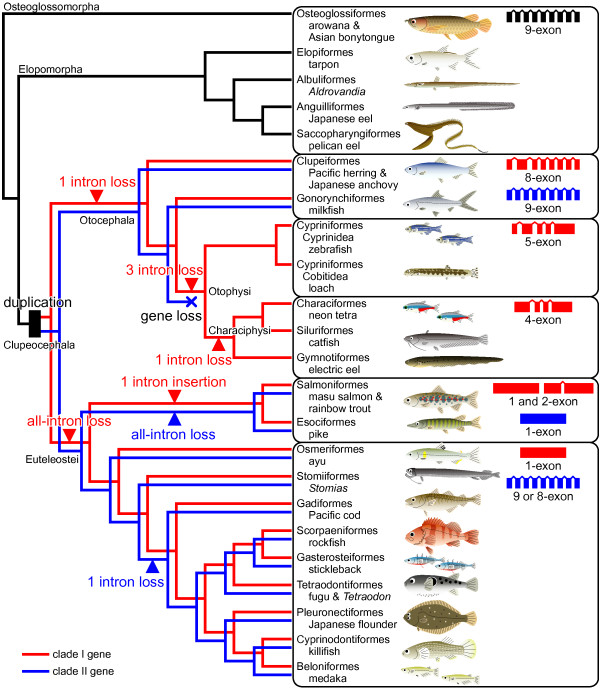
**Evolutionary pathway of teleostean hatching enzyme genes**. Duplication of hatching enzyme gene occurred in the ancestor of clupeocephalans (black square), and clupeocephalans possessed clade I genes (red line) and clade II genes (blue line). Clade II gene-loss occurred in the ancestor of otophysans. Intron-loss/insertion events are indicated at the lineage shown either with red (clade I genes) and blue (clade II genes) triangles. Resultant exon-intron structures are shown at the right with the same colors. The cladogram of teleostean relationships is based on the molecular phylogenetic relationships estimated using whole mitochondrial DNA sequences with some modifications [24-27].

An evolutionary pathway of exon-intron structural changes of hatching enzyme genes was deduced on the basis of the molecular phylogenetic relationships of teleosts estimated using whole mitochondrial DNA sequences (Figure 2). Osteoglossomorphs and elopomorphs possessed a single type of hatching enzyme gene. Gene duplication occurred after the branching off of clupeocephalans from the ancestor, and clupeocephalans then possessed two kinds of the genes, clade I and II genes. Then, the ancestor of otophysans lost the clade II genes. During the evolution of otocephalans, clade I genes lost their introns in a stepwise manner as one intron loss in the basal lineage of otocephalans, three more intron losses in the ancestor of otophysans, and an additional intron loss in the ancestor of characiphysans. During the evolution of euteleosts, all introns were lost from clade I genes, and one intron was newly insered into one of two clade I genes in the common ancestor of salmoniforms and esociforms. In contrast to the clade I genes, clade II genes did not undergo frequent structural change; nevertheless, one intron loss (8th intron) during the evolution of euteleosts and the loss of all introns in the common ancestor of salmoniforms and esociforms were observed.

### Why were introns lost?

Why did frequent intron losses occur during the evolution of hatching enzyme genes in Teleostei? We hypothesize that the intron loss events resulted from adaptation to efficient production of hatching enzyme according to the following information. It has been estimated to take 1 min to transcribe 1-1.5 kbp sequence and 3 min to remove an intron from the sequence of pre-mRNA [9]. Based on this information, we tentatively estimated the times for transcription of intron-possessing or intron-less hatching enzyme genes. According to the average length of exonic regions in hatching enzyme genes (1 kbp) and the average intron size (about 250 bp), the size of 8-intron genes is about 3 kbp and the size of intron-less gene is about 1 kbp. Therefore, an 8-intron gene would require 2-3 min for transcription and additional time (at least 3 min) for splicing introns. On the other hand, it would take less than 1 min to transcribe an intron-less gene. Indeed, the intron-less genes are well-known to tend to be rapidly expressed [7,38].

**Figure 3:**
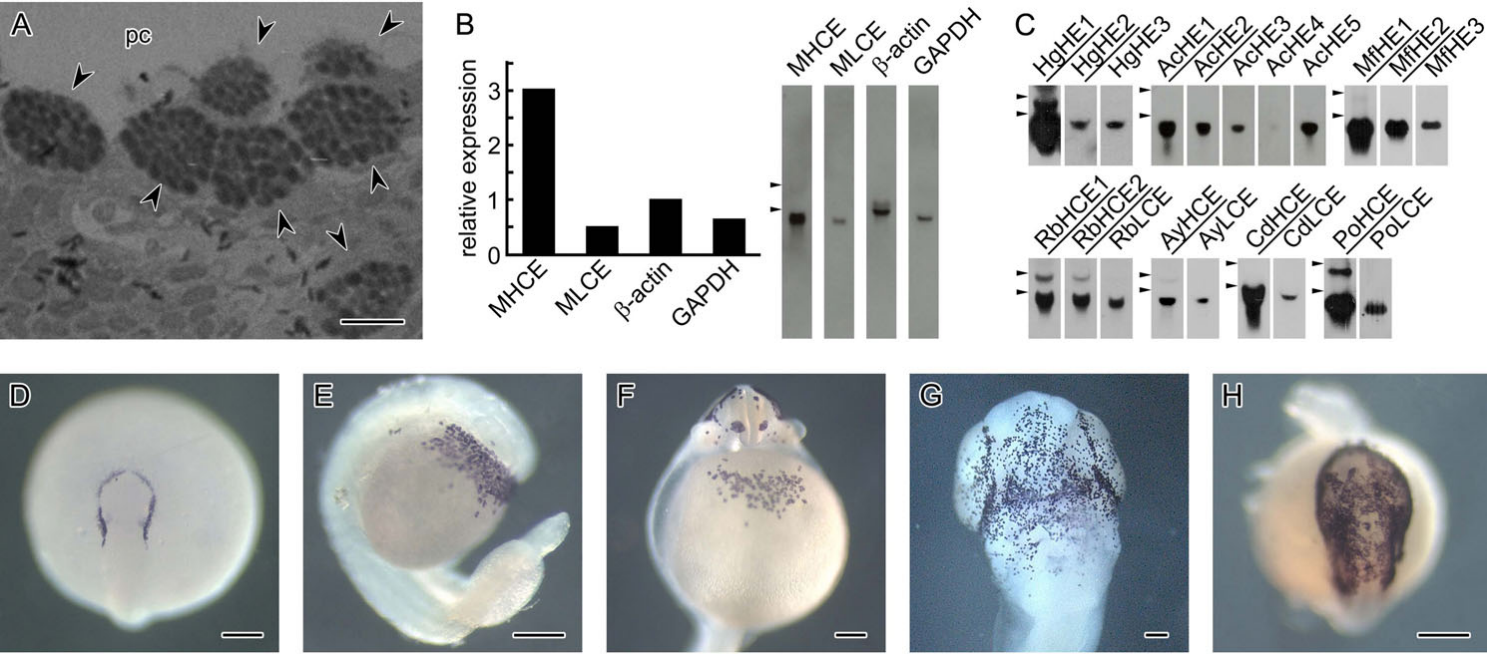
**Expression level of hatching enzyme genes and distribution of hatching gland cells (HGCs)**. (A) Comparison of the relative expression levels of hatching enzyme genes (*MHCE *and *MLCE*) and housekeeping genes (*β-actin *and *GAPDH*) in medaka embryos performed by Northern blot. The relative expression level of mRNA was estimated by densitometry and represented relative to an intensity of 1 for *β-actin*. (B) Expression analysis of hatching enzyme genes performed by Northern blot. Gene names are shown at the top of the lane, and clade I genes are underlined. Triangles indicate the positions of 28 S and 18 S rRNAs. (C) A section of the lower jaw of medaka embryo stained with toluidine blue. HGCs (arrowheads) are filled with many of zymogen granules. pc, pharyngeal cavity. Scale bar, 10 μm. (D-H) Distribution of HGCs were observed by whole-mount *in situ *hybridization using pre-hatching embryos of milkfish (D), loach (E), catfish (F), rainbow trout (G) and Pacific cod (H). Dorsal (D and H) and ventral (F and G) views of the head region, and lateral view (E). Scale bars, 200 μm.

We first examined whether or not hatching enzyme is more efficiently expressed than housekeeping genes in medaka embryos. As shown in Figure 3A, the expression level of the *MHCE *gene was three-fold higher than that of the housekeeping genes *β-actin *and *GAPDH*, while the level of *MLCE *gene was similar to those of the housekeeping genes. These expression levels were not as high as we expected. We further compared the expression level in a cell called hatching gland cell (HGC), where hatching enzyme genes were expressed. HGCs are distributed on the inner wall of the pharyngeal cavity in pre-hatching embryos of medaka [39], or the surface of the embryos of milkfish (Figure 3D), loach (Figure 3E), catfish (Figure 3F), rainbow trout (Figure 3G) and Pacific cod (Figure 3H). Hatching enzyme genes, unlike housekeeping genes, were expressed in restricted cells, indicating that the expression level of hatching enzyme genes in a cell is much higher than that of the housekeeping genes. The enzymes are accumulated in zymogen granules which are filled until immediately before hatching (Figure 3C) [40]. These results suggest that HGCs devote their substantial resources to synthesizing the hatching enzyme.

Next, we examined the expression levels between clade I and II genes in several fish species. Figure 3A indicates that expression of *MHCE *gene (clade I) is 6-times higher than that of *MLCE *gene (clade II) in medaka embryos. In addition, Northern analysis of 7 other fish species showed that clade I genes had a tendency to be more highly expressed than clade II genes (Figure 3B). Such differential expression reflected the relative amount of enzyme required for egg envelope digestion. At the time of medaka hatching, MHCE and MLCE cooperatively digest the egg envelope: MHCE swells the egg envelope by its proteolytic action, and MLCE digests the MHCE-swollen envelope completely [32,33,41]. *In vitro *experiments revealed that a large amount of MHCE was required to swell the envelope, while only a small amount of MLCE, 10-times less than that of MHCE, was enough to digest it [33,42]. This relationship has been also found in killifish [34]. These results suggest that clade I genes are required for production of a large amount of their proteins. Considering that a gene having a small number of introns is favorable for its high expression, clade I genes might have lost their introns during evolution so as to be highly expressed in HGCs.

### How were introns lost?

Currently, two main models are proposed for the mechanism of intron loss [11]: (1) deletion at the genome level [43]; and (2) homologous recombination between the genomic copy of a gene and the cDNA produced by the reverse transcription of its mature mRNA or partially spliced pre-mRNA [44]. Deletion under the first model does not always result in the exact removal of an intron region. In the hatching enzyme genes examined, we could not find any additional nucleotide insertions or deletions around the lost insertion sites in the mature enzyme-coding region. Such a precise intron deletion may be better explained by the second model involving homologous recombination. However, the evolutionary fixation of such intron deletion must be limited to the genes expressed in the germline. The hatching enzyme genes are expressed only during embryogenesis, and are never expressed in germ cells [21].

It is difficult to explain that six intron-loss events were generated in hatching enzyme genes only by homologous recombination, because it is hard to consider that the misexpression of hatching enzyme genes could occur so frequently in germ cells. There are some other examples of intron losses that do not seem to be mediated by reverse transcriptase (i.e., intron loss from genes expressed in non-germline, somatic cells, occurring without any nucleotide deletions or insertions [45]). An "unknown mechanism" by which introns are properly removed must exist. Although the intron-loss evolution of teleostean hatching enzyme genes may be a rare case under the special circumstances, future investigations of this phenomenon may reveal and improve the understanding of a new mechanism of intron loss.

## Conclusions

Ancestral hatching enzyme gene of teleosts is considered to be a single type of gene. After the branching off of clupeocephalans from the ancestor, duplication of hatching enzyme gene occurred. Consequently, the clupeocephalans possessed two kinds of hatching enzyme genes, called clade I and II genes. Clade I genes lost their introns frequently (1-8 intron losses), in contrast to the clade II genes (0-1 intron loss, with one exception showing 8 intron losses). When comparing expression level, "intron-lost genes" tend to be more highly expressed than "intron-conserving genes", suggesting that there might be a connection between the intron-loss evolution and the level of expression.

## Methods

### Fish

Embryo and adult fish samples were obtained from the following organizations: Japanese flounder *Paralichthys olivaceus *from National Center of Stock Enhancement, Fisheries Research Agency Miyako Station; milkfish *Chanos chanos *from Institute of Cellular and Organismic Biology of the Academia Sinica; loach *Misgurnus anguillicaudatus *from Graduate School of Fisheries Sciences, Hokkaido University; catfish *Silurus asotus *from Chiba-Prefectural Fisheries Research Center Fresh-Water Station; masu salmon *Oncorhynchus masou *from National Research Institute of Aquaculture; rainbow trout *Oncorhynchus mykiss *from Irikawa Trout Hatchery, Tokyo; Pacific cod *Gadus macrocephalus *from Toyama Prefectural Fisheries Research Institute Fisheries Resource Section; electric eel *Electrophorus electricus*, pike *Esox americanus, Stomias nebulosus*, *Aldrovandia affinis *and pelican eel *Eurypharynx pelecanoides *from Atmosphere and Ocean Research Institute, The University of Tokyo; arowana *Osteoglossum bicirrhosum*, Asian bonytongue *Scleropages formosus*, tarpon *Megalops cyprinoides *and neon tetra *Paracheirodon innesi *from dealer.

### Cloning of hatching enzyme cDNAs and their genomic sequences

Teleostean hatching enzyme genes reported previously were multi-copy genes [12]. To clone all kinds of hatching enzyme genes, we designed four sets of PCR primers from the regions conserved in the cloned genes [21]. Using these primers, we isolated the full length hatching enzyme cDNAs from RNAs of the pre-hatching embryos of 6 species by RT-PCR and RACE PCR. After their genomic genes were isolated, the exon-intron boundaries of the genomic DNAs were determined by comparison with the sequences of cDNAs.

In cases that we could not obtain embryos, we cloned hatching enzyme genes by PCR directly from genomic DNAs of the adult fishes. The exon-intron boundaries of the cloned genes were also confirmed based on similarities to the sequence of closely-related species. The method of direct cloning from the genomic DNAs is as follows. Five primers, four forward (F1 to 4) primers and one reverse (R1) primer, were designed from the conserved sequences of hatching enzyme cDNAs [21]. From the fragments, we further constructed forward and reverse gene-specific primers (GSPs). Next, 5'- and 3'-portions of the gene were separately extended using the forward GSP and R2 primer designed from the 3'-end conserved in the cloned cDNAs, or using the reverse GSP and F5 primer designed from the 5'-end conserved in the cloned cDNAs. The sequences of F1-5 and R1-2 primers are shown below, and the positions are shown in Additional file 2.

F1: 5'-RVMVRMTGYYTiTGGARSAARDVYBC-3'

F2: 5'-ARACMTGCATTCGYTTYRTBYCHHG-3'

F3: 5'-YRYYCAGCAYGAGMYBMDYCAYGCiCTSGG-3'

F4: 5'-HTTCYAiCAYGARCAHDYHAGRAGCGAYCG-3'

F5: 5'-TBCWRVYBCTGBTVBTBRGMHTYTCiYWRGC-3'

R1: 5'-TTCCATARTGCATSABVGARSHRTAGTCRTA-3'

R2: 5'-RCAKYYRTABAKHiKVTTGATYCYSARRATRTC-3'

As previously described [12], most of the hatching enzyme genes were predicted to be multi-copy genes forming clusters with short inter-genic regions. Therefore, the inter-genic region between hatching enzyme genes was amplified using GSPs. In some cases, the 5' or 3' regions of the gene were extended by the ACP-PCR method using DNA Walking SpeedUp Premix Kit (Seegene Inc., Seoul, Korea). By the combined use of the above PCR methods, the full-length genes including their 5'-upstream and 3'-downstream regions were cloned. The sequences thus cloned included the hatching enzyme genes and their paralogous genes. As reported previously [21], hatching enzyme genes are specifically expressed in pre-hatching embryos, while their paralogous genes such as nephrosin gene are mainly expressed in adult internal organs. We discriminated hatching enzyme genes from their paralogous genes based on the phylogenetic analysis and whole-mount *in situ *hybridization. The names of the cloned hatching enzyme genes are listed in Additional file 1.

### Phylogenetic analysis

A codon-based alignment of nucleotide sequences of mature enzyme portions was made using the Clustal X program [46] and the CodonAlign 2.0 program [47]. Data were partitioned into the first, second and third codon positions. A general time reversible (GTR) + I + Γ was selected as the best fitting model using Kakusan4 [48]. Maximum likelihood (ML) analysis was conducted with RAxML version 7.2.6 [49], using the GTR + I + Γ model. We reconstructed an ML tree, simultaneously conducting bootstrap analysis for the best-scoring topology with 1,000 replicates.

The program Notung version 2.6 [28,29] was employed to reconcile the hatching enzyme gene tree with teleostean species tree. Notung mapped duplication and loss events of the genes onto branches of the species tree by reconstructing ancestral states according to the parsimony method. The topology of the species tree was obtained from the molecular phylogenetic tree estimated using whole mitochondrial DNA sequences [24-27].

### Whole-mount *in situ *hybridization

Whole-mount *in situ *hybridization was performed according to the method described previously [50]. The digoxigenin (DIG)-labeled RNA probes were synthesized from hatching enzyme cDNAs of milkfish, loach, catfish, Pacific cod and rainbow trout, and were hybridized to pre-hatching embryos.

### Northern blot analysis

For comparison of the expression levels of medaka hatching enzyme genes (*MHCE *and *MLCE*) and housekeeping genes (*β-actin *and *GAPDH*), total RNA was extracted from stage 30 embryos. Four μg of the RNAs were electrophoresed on 1% formaldehyde-agarose gel, and then transferred to nylon membrane (Hybond N, Amersham, Piscataway, NJ, USA). cDNAs of *MHCE*, *MLCE*, *β-actin *and *GAPDH *were used to synthesize DIG-labeled PCR probe. Each probe size was adjusted to 816 bp and the labeling efficiency was checked by dot-blot analysis. Hybridization was performed using the same protocol as described previously [50]. Images were analyzed with ImageQuant software (Molecular Dynamic, Sunnyvale, CA) for semi-quantitative assessment of the expression level.

## Accession Numbers

The nucleotide sequence data reported in the present paper will appear in the DDBJ/EMBL/GenBank nucleotide sequence databases with accession number [AB480003-AB480032].

## Abbreviations

HE: hatching enzyme; HCE: high choriolytic enzyme; LCE: low choriolytic enzyme; HGC: hatching gland cell

## Authors' contributions

MK, JH, MM, MN, II and SY designed the study. MK carried out data collection, data analysis, and manuscript preparation. MN, II and SY supervised and finalized the manuscript. All authors read and approved the final manuscript.

## Supplementary Material

Additional file 1 Teleostean species examined in this study and the names of hatching enzyme genes Click here for file

Additional file 2 **A multiple alignment of amino acid sequences deduced from teleostean hatching enzyme genes**. All hatching enzymes were composed of a signal sequence (putative cleavage sites are shown as white triangle), a pro-sequence, and a mature enzyme sequence (the N-terminals are shown as black triangle). The mature enzyme portion possesses two active site consensus sequences for astacin family metallo-proteases, HExxHxxGFxHExxRxDR (Zn-binding site, indicated in dark gray) and SxMHY (methionine turn, indicated in light gray). In addition, six conserved cysteine residues are shaded in black. Red lines indicate the intron insertion sites. Identical residues are boxed. Dashes, asterisks and "X"s represent gaps, stop codons and unidentified amino acid residues, respectively. Arrows at the bottom indicate sites of primers designed for amplification of hatching enzyme gene fragments. Click here for file
