# Supplementary material for: Intron-loss evolution of hatching enzyme genes in Teleostei
Source: BMC Evol Biol. 2010 Aug 27;10:260. doi: 10.1186/1471-2148-10-260 (PMC2939575; doi:10.1186/1471-2148-10-260)
Supplement: Additional file 1 — Teleostean species examined in this study and the names of hatching enzyme genes [file 1471-2148-10-260-S1.DOC]

| Classification* | Species | Name in this study | Gene name: Accession No. | Source | Reference |
| --- | --- | --- | --- | --- | --- |
| Osteoglossomorpha |  |  |  |  |  |
| Osteoglossiformes | *Osteoglossum bicirrhosum*  *Scleropages formosus* | arowana  Asian bonytongue | AwHE: [AB276000]  BtHE: [AB480003] | genomic DNA  genomic DNA | This study  This study |
| Elopomorpha |  |  |  |  |  |
| Elopiformes | *Megalops cyprinoides* | tarpon | TpHE: [AB480004] | genomic DNA | This study |
| Albuliformes | *Aldrovandia affinis* | *Aldrovandia* | AaHE1/2: [AB480005/6] | genomic DNA | This study |
| Anguilliformes | *Anguilla japonica* | Japanese eel | EHE4/7: [AB071423/5] | mRNA | Hiroi et al. 2004 |
| Saccopharyngiformes | *Eurypharynx pelecanoides* | pelican eel | PeHE1/2: [AB480007/8] | genomic DNA | This study |
| Otocephala |  |  |  |  |  |
| Clupeiformes | *Clupea pallasii*  *Engraulis japonicus* | Pacific herring  Japanese anchovy | HgHE1-3: [AB433584-6]  AcHE1-5: [AB433587-91] | mRNA  mRNA | Kawaguchi et al. 2009  Kawaguchi et al. 2009 |
| Gonorynchiformes | *Chanos chanos* | milkfish | MfHE1-3: [AB480009-11] | mRNA | This study |
| Cypriniformes | *Danio rerio*  *Misgurnus anguillicaudatus* | zebrafish  loach | ZHE1/2: [AB175621/0]  LoHE1/2: [AB480012/3] | mRNA  mRNA | Inohaya et al. 1997  This study |
| Characiformes | *Paracheirodon innesi* | neon tetra | NeHE1/2: [AB480014/5] | genomic DNA | This study |
| Siluriformes | *Silurus asotus* | catfish | CfHE1-3: [AB480016-8] | mRNA | This study |
| Gymnotiformes | *Electrophorus electricus* | electric eel | EeHE1/2: [AB480019/20] | genomic DNA | This study |
| Euteleostei |  |  |  |  |  |
| Salmoniformes | *Oncorhynchus masou*  *Oncorhynchus mykiss* | masu salmon  rainbow trout | MsHCE1/2: [AB175619/8]  MsLCE: [AB480021]  RbHCE1/2: [AB480022/3]  RbLCE: [AB480024] | mRNA  genomic DNA  mRNA  mRNA | Inohaya et al. 1997  This study  This study  This study |
| Esociformes | *Esox americanus* | pike | PkHCE1/2: [AB480025/6]  PkLCE: [AB480027] | genomic DNA  genomic DNA | This study  This study |
| Osmeriformes | *Plecoglossus altivelis* | ayu | AyHCE: [AB256940]  AyLCE: [AB256942] | mRNA  mRNA | Kawaguchi et al. 2006  Kawaguchi et al. 2006 |
| Stomiiformes | *Stomias nebulosus* | *Stomias* | SnHCE: [AB480028] | genomic DNA | This study |
| Gadiformes | *Gadus macrocephalus* | Pacific cod | CdHCE: [AB480029]  CdLCE: [AB480030] | mRNA  mRNA | This study  This study |
| Scorpaeniformes | *Helicolenus hilgendorfi* | rockfish | HhHCE1/2: [AB353102/3]  HhLCE: [AB353104] | genomic DNA  genomic DNA | Kawaguchi et al. 2008  Kawaguchi et al. 2008 |
| Gasterosteiformes | *Gasterosteus aculeatus* | stickleback | GaHCE1/2: [AB353108/9]  GaLCE: [AB353110] | mRNA  mRNA | Kawaguchi et al. 2008  Kawaguchi et al. 2008 |
| Tetraodontiformes | *Takifugu rubripes*  *Tetraodon nigroviridis* | fugu  *Tetraodon* | FgHCE: [AB246041]  FgLCE: [AB246042]  TnHCE: [AB246043]  TnLCE: [AB246044] | mRNA  mRNA  genomic DNA  genomic DNA | Kawaguchi et al. 2007  Kawaguchi et al. 2007  Kawaguchi et al. 2007  Kawaguchi et al. 2007 |
| Pleuronectiformes | *Paralichthys olivaceus* | Japanese flounder | PoHCE: [AB480031]  PoLCE: [AB480032] | mRNA  mRNA | This study  This study |
| Cyprinodontiformes | *Fundulus heteroclitus* | killifish | FHCE: [AB210813]  FLCE: [AB210814] | mRNA  mRNA | Kawaguchi et al. 2005  Kawaguchi et al. 2005 |
| Beloniformes | *Oryzias latipes* | medaka | MHCE: [M96170]  MLCE: [M96169] | mRNA  mRNA | Yasumasu et al. 1992  Yasumasu et al. 1992 |

*Classification of species followed Nelson (2006)
