# Supplementary material for: Intron-loss evolution of hatching enzyme genes in Teleostei
Source: BMC Evol Biol. 2010 Aug 27;10:260. doi: 10.1186/1471-2148-10-260 (PMC2939575; doi:10.1186/1471-2148-10-260)
Supplement: Additional file 2 — A multiple alignment of amino acid sequences deduced from teleostean hatching enzyme genes. All hatching enzymes were composed of a signal sequence (putative cleavage sites are shown as white triangle), a pro-sequence, and a mature enzyme sequence (the N-terminals are shown as black triangle). The mature enzyme portion possesses two active site consensus sequences for astacin family metallo-proteases, HExxHxxGFxHExxRxDR (Zn-binding site, indicated in dark gray) and SxMHY (methionine turn, indicated in light gray). In addition, six conserved cysteine residues are shaded in black. Red lines indicate the intron insertion sites. Identical residues are boxed. Dashes, asterisks and "X"s represent gaps, stop codons and unidentified amino acid residues, respectively. Arrows at the bottom indicate sites of primers designed for amplification of hatching enzyme gene fragments. [file 1471-2148-10-260-S2.PDF]

```
Osteoglossomorpha & Elopomorpha HE
AwHE     1:MEGK------LTCTILALLLSFSQANP-----LMDLQGEDD----------------------------- 30
BtHE     1:MEGQ------LTCTILALLLSFSQANP-----LMDLQIEDD----------------------------- 30
TpHE     1:MDHR------LVSTVLALLLSLSQAHP-----LMKLESGEV----------------------------- 30
AaHE1    1:MDHK------LLATILALLLSLSQVHH-----LVELGNEDG----------------------------- 30
AaHE2    1:MDHE------LLATILALLLSLSQAHH-----LVELGNEDG----------------------------- 30
EHE7     1:MDQR------LISTILALLLSLSLAHH-----VEDLESEDA----------------------------- 30
PeHE1    1:MDQR------LIYTILALLLSLSQAQH-----FEDLESEDE----------------------------- 30
EHE4     1:MDQR------LISTILALLLSLSQAHP-----LVDLGSEADDSPLIS----------------------- 36
PeHE2    1:MDQR------LISTILALLLSLSQAHP-----LVDLGSEADDSPLIS----------------------- 36
Otocephala clade II
HgHE3    1:MDRK------LAC-ILVLIVGLSRALS-----QMDQDMSSPEE--------------------------- 31
AcHE3    1:MSGK------VVS-MLTLLLALSHALA-----QESQDNMKEQSAEEKKSRALLYGSYELDTSNDQEEKQE 58
AcHE4    1:MGRK-------LAAILALLLAVSQALAQAEESQEDQYDLEDEEEEMEVSDEFLDEKQV------------ 51
AcHE5    1:MGGK----LASI-LALALLLSISHSAH-----VGDKNDRHETNESSDISSRILNAHKATYGLEDELLHEQ 60
MfHE3    1:MDHR------LVSAVLILLLGLGQALS----STEQPSVEE------------------------------ 30
Euteleostei clade II
AyLCE    1:MDPSMIDNSKMAMFLLLVGLGLSQAHP-----AE-----KE----------------------------- 31
CdLCE    1:MAAR-----TITLGLLLFLLAVSQAQ-----EIKEGEEQQSNVTG------------------------- 35
FgLCE    1:MVGA------TVSLLLLLL-GLCTAH-------KPLQTDKV----------------------------- 27
TnLCE    1:MLGT------TVSLLLLLL-GFCSAD-----DDNLHEHEKV----------------------------- 29
GaLCE    1:MDLR-------AAFPLLLVLGLGCAE-----QGGDHGGANE----------------------------- 29
MLCE     1:MDLL-----AKASVLLLLLLSLSNAQ-----TDNMEEAENG----------------------------- 31
FLCE     1:MDLQ-----ARALLLLLLLSAVCNAY-----PTDNYKADDE----------------------------- 31
HhLCE    1:MDPR-----TTASLLVLLLLGLCNAH-----EGNDHDANNV----------------------------- 31
PoLCE    1:MDFK-----TSSSLLLLLLLGFCNAH-----QGNDQGADDD----------------------------- 31
MsLCE    1:MAHR-----PTLNLLLLLL-GLSQAS------GNEVHD---DHVS------------------------- 30
RbLCE    1:MAHR-----PTLNLLLLLL-GLSQAS------GNEVHD-EPDHVS------------------------- 32
PkLCE    1:MDHR-----PTLGLVLLVL-GLSLAS------GNDAQIYKREYSS------------------------- 33
Otocephala clade I
HgHE1    1:MDLR-----ASNSLLVLLL-GLSKALP-----VMEDSDENE----------------------------- 30
HgHE2    1:MELR-----ASLSLLVLLL-GLCEAMP-----FMSGVDYD------------------------------ 29
AcHE1    1:MDLR-----ASISLLLLLLIGLSQALP-----VTEDNDQ---HDFSE----------------------- 34
AcHE2    1:MDLR-----ASISLLLLLLIGLSHALP-----ATEEDAEDESHAFSDEPEDSDVTTTETPETSNYADEE- 59
MfHE1    1:MDLR-----ASLSLLVLLM-GLSQAVH-----LMENE-KDN----------------------------- 29
MfHE2    1:MNLR-----ASLSLLVLLL-GLSQAIP-----VMENEIEDN----------------------------- 30
ZHE1     1:MDIR-----ASLSILLLLF-GLSQASP-----LREFEAVF------------------------------ 29
ZHE2     1:MDPK-----ISLSIQLLLV-GISLAAP-----VGEYDNSN------------------------------ 29
LoHE1    1:MDSR-----ASFSILMLLV-GFSQAFI-----LPEQRLAD------------------------------ 29
LoHE2    1:MDPR-----ASFSILMLLVR-FSQAFI-----LPGQKLADT----------------------------- 30
NeHE1    1:MEPT-----ASLSILALLMLGFTQAVP-----LIESQHV------------------------------- 29
NeHE2    1:MEPI-----ASLSILALLILGFTQAVP-----LTELQHV------------------------------- 29
EeHE1    1:MEPR-----ASFSILALLL-GLSQALP-----TTEPQHV------------------------------- 28
EeHE2    1:MEPR-----ASYSILALLL-GLSQALP-----TTEPQYV------------------------------- 28
CfHE1    1:MMSR-----ASLSILALLL-GFSQALH-----LIQPEQE------------------------------- 28
CfHE2    1:MESR-----ASLPILALLL-GFSQALY-----LTQPDGE------------------------------- 28
CfHE3    1:MESR-----ASLFILVLLL-GFSQALY-----LTQPDGE------------------------------- 28
Euteleostei clade I
AyHCE    1:MKME---ISTSFSLLLILL-GLSHAVP-----LLDENPK--DHRK------------------------- 34
SnHCE    1:MK-----TSPTFSLLLLLLLGFSQASP-----LLD---VLADDV-------------------------- 31
CdHCE    1:MK-------TCLSLLLLLL-GLCQAQP-----LVGEES-VADVLEDNEDMDIITEILTSNNDSNEILQKG 56
FgHCE    1:MT-------PYVSLLLLLLLGLSQAQP-----VKEEGSNAVD---------------------------- 30
TnHCE    1:MT-------PCVSLLLLLL-GLSQAQP-----VKEEGGNAVG---------------------------- 29
GaHCE1   1:MT-------PTASLLLLLLLGFSEAYP-----LVDEGSEAND---------------------------- 30
GaHCE2   1:MT-------PTASLLLLLLLGFSEAYP-----LVDEGSEAND---------------------------- 30
MHCE     1:MN-----LAPSTCLLLLFLLDIAQALP-----VWDEEGHEEGHEEG------------------------ 36
FHCE     1:MI-------SSTSLLLLLLLGFSRALP-----LQEEGDQEEEKEEG------------------------ 34
HhHCE1   1:MK-----MTPSASLLLLLLFGLSQALP-----LQEEG-GEEEEV-------------------------- 33
HhHCE2   1:MT-------PSASLLLLLLLSLSQAHP-----LMEEGSGDEAQV-------------------------- 32
PoHCE    1:MT-------PTVSLLLLLLLGLSQAQP-----LQEEVIAEDVP--------------------------- 31
MsHCE2   1:ME-----QSLSLSILLLVL-GLSQAQM--------DHSGPAMWIDHLEAV-------------------- 36
RbHCE2   1:ME-----QSPSLTILLLVL-GLSQAQM--------DHSGPAMWIDHLEAV-------------------- 36
PkHCE2   1:ME-----LTTSLNLLVLL--GLARALP--------TR------AEHPKAM-------------------- 29
MsHCE1   1:ME-----QSPSLTLLLLLL-GLSQANP-----LMEDGSGPEILTDNPEAV-------------------- 39
RbHCE1   1:ME-----QSPSLTLLLLLLLGLSQANP-----LMEDGSGPEILTDNPEAV-------------------- 40
PkHCE1   1:ME-----LIPSVSLLLLLV-GTSQASP-----VM-------KQVDGPEIS-------------------- 32
                        F5
```

```
Osteoglossomorpha & Elopomorpha HE
AwHE     31:----------------VQPEDPEHV-DMTTRILQMNNGSS----QMLLQGDLLVPNTRNAMICY--LNRCL 78
BtHE     31:----------------VQPEDPEHV-DITTRILQTNNGSS----QVLLEGDLLVLNTRNAMICY--LNSCL 78
TpHE     31:----------------IQIEDPEDL-DITTRILESNNGSS----QMLMEGDLIVSNTRNALNCW--HRKCL 78
AaHE1    31:----------------IQTEDHEDL-DITTRILQSNNGSR----EMLMEGDLVVSNTRNALNCR--NKQCL 78
AaHE2    31:----------------IQTEDHEDL-DITTRILQSNNGSR----EMLMEGDLVVSNTRNALNCR--NKQCL 78
EHE7     31:----------------NKIEDPDDL-DITTRIMQSNNGSI----EMLMEGDLLVSNTRNAMKCR--NNRCL 78
PeHE1    31:----------------IQMED---L-DITTRILKSNNGSS----EMLMEGDLVVSKTRNAMKCR--NNRCL 75
EHE4     37:----------------IQIEDPDDV-DITTSILQSNNGSS----EILMEGDLIVSNTRNAMKCW--NNQCL 84
PeHE2    37:----------------IQIEDPDDV-DITTTILQSNNGSS----EMLMEGDLIVSNTRNAMKCW--NNQCL 84
Otocephala clade II
HgHE3    32:---------------------MDEAVDISSRILDANRGIS----RTLMEGDVAMPRSRNAMKCY-SFYDCR 76
AcHE3    59:---------------------TGEEPDITSRIMEANKRIYGYYAGFLMEGDVAVPRGRNARMCA-DNKPCL 107
AcHE4    52:---------------RFLDEAEKEGSMTGDILEANKWLF----SYLSEGDVAVPTGRNAMKC----TNCY 98
AcHE5    61:SRPDQDQHVKIHGEYVSTEETNESSDISSRILEANKDIS----DHLMEGDVALPRTRNAMRCL--YYDCK 124
MfHE3    31:--------------------DHEKL-DITSVILEANKGIS----HMMMEGDVVIAKTRNAMKCW--ADYCK 74
Euteleostei clade II
AyLCE    32:--------------LLEVHMEAHTFEKSNQTILQINKEIN----ELLLEGDIATPKNRNARKCAANAYTCL 84
CdLCE    36:----------------------TVA--VGDTILMMNSGSP----EFLIDGDVLISRKRSAMKCYSQSFSCL 78
FgLCE    28:--------------------DPTEKEDITTTILRMNNGSS----EFLLEGDLYIPKTRNVMKCLNKAFNCL 74
TnLCE    30:--------------------DPTKGEDITSTILRMNNESA----DFLLEGDLFIPKKRNAMKCLNDAFNCL 76
GaLCE    30:-------------------------EDLTTVIMKMNKGSD----GYLLEGDMLIQTQRNAIRCSNPRYSCL 71
MLCE     32:--------------SSKEEIDESELEDVSSIIFRMNNNSM----EELLEGDLVLPKTRNAMKCFGAPDSCR 84
FLCE     32:---------------------NSEKEDITTTILRMNNGSA----DMLFEGDVFVPRSRTAKKCLDPRYSCF 77
HhLCE    32:--------------------DDSEMEDLTSTILRMNNGSS----DFLLEGDVLIPRTRNAMKCFNQAYTCL 78
PoLCE    32:-----------------TSVDSSDVEDITATILRMNNGST----DFLMEGDVLIPRTRNAMKCFAKSYTCL 81
MsLCE    31:---------------------------ITSAILESNNGTN----ELLLEGDILAPRTRNAMKCFSSQYSCL 70
RbLCE    33:---------------------------ITSAILESNNGTN----ELLLEGDILAPRTRNAMKCFSSQYSCL 72
PkLCE    34:---------------------------ITSIILNSNHGTS----ELLLEGDILVPKTRNAMKCYKNNGRCF 73
Otocephala clade I
HgHE1    31:----------------IFLEESDSV-DLTTQILSTNNASS----ELLVEGDLVVPRTRNAMACW--NNQCL 78
HgHE2    30:----------------IFTHEPGDT-DLTTQILTANKDSE----ELLVEGDVAVQLQRNAVSC----FFCK 75
AcHE1    35:------------------DETEEDY-DLTEQILGTNNASK----ELLLEGDLVVPKTRNALSCF--KNQCL 80
AcHE2    60:-----------------TDPEEPDY-DLTTQILDTNNAST----EFLMEGDLVAPKTRNALACW--NNQCL 106
MfHE1    30:----------------FLLEEPETV-DITTRILDSNNAST----ELLVEGDLIVPRTRNALNCW--NNYCL 77
MfHE2    31:----------------FLPEEEETV-DMTARILDSNKRYS----EVLVEGDLVVPKTRTAVNCW--NNHCL 78
ZHE1     30:------------------VSEPETV-DITTQILETNKGSS----EVLFEGDVVLPKNRNALICE--DKSCF 75
ZHE2     30:-----------------GIETPQNV-DITT-LLETNKGSS----RRLIEGDMLYPQTRNALVCG--NNNCF 75
LoHE1    30:-----------------LLTEPEDV-DITTRILETNNGST----EILLEGDLLFPKTRNALYCL--TNNCF 76
LoHE2    31:-----------------LVSEPENV-DITTKILETNNGSA----EILLEGDLVFPKTRNALYCL--NNNCF 77
NeHE1    30:--------------------------DITTRILSTNNGSR----ELLVEGDIVLPRTRNALVCP--SNNCF 68
NeHE2    30:--------------------------DITTRILSTNNGSR----ELLVEGDIVLPRTRNALVCP--SNNCF 68
EeHE1    29:--------------------------DIISRIITINNGSS----EMLVEGDILLPETRNSLVCP--SNKCF 67
EeHE2    29:--------------------------DITSRIITINNGSS----EMLVEGDILLPETRNSLVCP--SNKCF 67
CfHE1    29:--------------------------DFTSKILDVNHGSS----EPLLEGDILLPGIKNALVCP--DGSCF 67
CfHE2    29:--------------------------DITTRILTINKGSS----ELLLEGDILAKRSRNALVCL--YNNCF 67
CfHE3    29:--------------------------EITTRILTINKGSS----ELLLEGDILAKSSRNALVCL-YNNNCF 68
Euteleostei clade I
AyHCE    35:------------------------L-SIGS-ILTTNNQSN----EHPLEGDLVAPKTRNAMRCY-QGDECK 74
SnHCE    32:-------------------QDDDDV-DISSMILNTNNGSS----EMLLEGDMVLPRTRNAMTCY--NDYCR 76
CdHCE    57:DLSQAQPLVGEESVDDILEDNEDM-DISTRILISNNGSN----EILLEGDLLLPTTRNAMKCF--RNNCL 119
FgHCE    31:--------------------EEAPV-DISTRILSSNNGSK----QILLEGDLVAPKSRNAMKCW--SQSCL 74
TnHCE    30:--------------------EDDPV-DISAKILSSNNGSQ----EILLEGDMVAPKNINAMKCW--SQSCL 73
GaHCE1   31:-----------------QMDEDDSI-DITTRILNSNNGSN----EMLLEGDVLVPTTRNAMKCF--YQQCL 77
GaHCE2   31:-----------------QMDEDDSI-DITTRILNSNNGSN----EMLLEGDVLVPTTRNAMKCF--YQQCL 77
MHCE     37:-------------------DGDDFV-DITTRILTSNNNTD----QLLLEGDLVAPTNRNAMKCW--SSSCF 81
FHCE     35:---------------------ADTV-DMTTRILTANNASD----EMLLEGDIMLPKSRNAMKCW--YNSCV 77
HhHCE1   34:---------------------PDTV-DITTRILTSNNGTN----EILLEGDLLVPRTRNAMKCW--SQDCL 76
HhHCE2   33:-------------------DEDDTV-DISTRILTSNNATD----EILLEGDMLVPRTRNAIRCW--YQSCM 77
PoHCE    32:---------------------DDTM-DITTRILTSNNATD----EILLEGDLLAPRTRNAMMCW--SQSCQ 74
MsHCE2   37:--------------------------DITERILTANNGSD----EMLMEGDLVVPRTRNAMQCMQGGNSCL 77
RbHCE2   37:--------------------------DITERILTANNGSD----EMLMEGDLVVPRTRNAMQCMQGGNSCL 77
PkHCE2   30:--------------------------DYTERILTANSDSD----EILMEGDLVIPRTRSAMVCRQGENNCL 70
MsHCE1   40:--------------------------DITERILTTNNGSS----QFLLEGDMVAPTTRNAMICY--SAGCF 78
RbHCE1   41:--------------------------DITERILTTNNGSS----QFLLEGDMVAPTTRNAMICY--SAGCF 79
PkHCE1   33:---------------------------ITKTILTINNGSS----KFLLEGDIVSPTTRNAMICY--SSGCF 70
                                                                               F1
```

```
Osteoglossomorpha & Elopomorpha HE
AwHE     79:WIKSADGLVQVPYVLS-SDFSAVQQRLIESAMMAFSSFTCVHFVRRT-TQSDYISIENLDGCYSVLGRTG 146
BtHE     79:WKKSANGLVEVPYVLS-NDFSNVQKQLIESAMRDFSSLTCIRFVLHG-TQSDYISIENLDGCYSVLGRTG 146
TpHE     79:WRKSSNGLVEVPYTVS-DDFSYFEKWRIEYAMNTFHTRTCIRFVPRQ-SQSDYISIESKDGCYSYLGRTG 146
AaHE1    79:WKKSSNGLVEVPYIVS-NDFSCGEKQKIENAMKTFNNETCIRFVPRV-SQSDYISIENRDGCYSYLGRTG 146
AaHE2    79:WKKSSNGLVEVPYIVS-NDFSYGEKQKIENAMKTFNTETCIHFVPWF-SQSDYISIENRDGCYSSLGRTG 146
EHE7     79:WKKSSNGLVEVPYTVS-RQFSYYQRKRIVKAMKTFNTQTCIRFVPRS-RQRDYISIKSRGGCYSYLGRTG 146
PeHE1    76:WKKSSNGLVIVPYTIC-RVFINSQRKKIENAMKIFKNETCIRFVPRS-NQKDYISIQSKGGCYSYLGRTG 143
EHE4     85:WRKSSDGLVEVPYTVS-NEFSYHKKRIENAMKTFNTETCIRFVPRS-SQRDFISIESRDGCYSYLGRTG 152
PeHE2    85:WKKSSDGLVEVPYTVS-HDFPYYQRERIENAMKTFNTETCIRFVPRS-SQRDFISIEDRDGCYSYLGRTG 152
Otocephala clade II
HgHE3    77:WEKSSTGLVEVPYNID-DYFLSHEKVTIEKAMNTFHEKTCIRFVPYT-TQVHHLSIESKSGCYSSVGRTG 144
AcHE3   108:WEKSPSGLVRVPYRIA-YGFSRAKRGIIKKAMETFHQKTCIRFVPQLPHEMTFLFIESREGCWSYVGKRG 176
AcHE4    99:WEKAASGFVEVPYRLS-KNYSYRQKKLIKRAMRVFHKNTCIRFVPHT-SQSDYIDIMSKPGCWSYIGRIG 166
AcHE5   125:WPKSRSGLVEVPYNIS-DYYYASEKRTIERAMAALHEKTCVRFVPHT-NQLHYLSIESKTGCWSLVGRIG 192
MfHE3    75:WEKSSSGLVEVPYTIS-DYFYDYEKAKIKRAMRSFNEKTCIRFVPRT-GQTNYISIESKTGCWSNLRRTG 142
Euteleostei clade II
AyLCE    85:WKKSPNGKVEVPYVID-RLYQENEKNYIEYSMRDFAVKTCVRFVPRQ-DQDMYLHIVPKTGCFSGIGCYG 152
CdLCE    79:WPMSDNGKVEIPFTIS-DQYTGDEEAVILKAFEGFHSETCIRFIPRK-TQRMYLQFKSLFGCFSSVGRIG 146
FgLCE    75:WPKSSDGKVWIPYIIS-DKYDAEENDAIVTAMKDFHGKTCIRFRPRK-EERMYLSFQPRHGCFSAMGRVG 142
TnLCE    77:WPKSSDGKVWLPYLIS-EKYDQDEVDTILKALKDFHTKTCIRFKPRQ-GERMYLSFEPKHGCFSAMGRVG 144
GaLCE    72:WQKGASGYVEVPFEIS-NAYDNTDRNAIFTAMNEFKAKTCIRFVPRQ-REIAYLSIEPRAGCFSGVGRIG 139
MLCE     85:WPKSSNGIVKVPYVVS-DNYENSDEKETIRNAMKEFAEKTCIHFVPRN-NERAYLSLEPRFGCKSMMGYVG 152
FLCE     78:WPKSSNGNVEIPFVLS-DEYDHNEKNQILKAMKGFEGRTCIRFVRHR-GERAYLSIESKFGCFSLMGRSG 145
HhLCE    79:WRKSANGNVEIPFLIS-QKYDDAERSEILGAMKDFECKTCIRFIPRK-TQRAYLSVEPRSGCSSLLGTTG 146
PoLCE    82:WPKSANGNVEVPFLLS-EKYDDSEKREIVTALKDLEWKTCIRFVPRI-RQRAYLSFEPRYGCASLLGHVG 149
MsLCE    71:WKKSSDGLVYVPYILS-AVYSSLEVETIETAMKYFHGKTCIRFIPRK-TQTAYLDIQSSGGCFGTMGTVG 138
RbLCE    73:WKKSTDGLVYVPYILS-AVYSSLEVETIETAMKYFHGKTCIRFIPRK-TQTAYLDIQSSGGCFGTMGTVG 140
PkLCE    74:WMKSADNFVYVPYVIG-DEYSSDQVETIIMAMQSFHGKTCIRFIPRA-QESAYLQIESRGGCFSSMGRVG 141
Otocephala clade I
HgHE1    79:WKKSSNGKVEVPYTVN-SQFSSSDKSKIQNAMATFNRKTCVQFVARS-TQRDYISIENRDGCFSSLGRTG 146
HgHE2    76:WKKSANGLVEVPMTIS-PDFSFYDRMKIEKAMLTFHEQTCVRFVPRS-TEIDYLSLENGHGCHSYIGRAG 143
AcHE1    81:WRKSADGTVKVPYTIK-SYFSTSEKNKMKRAMEVFHKKTCIRFVPQS-TETAYVAIESRDGCFSYLGRTG 148
AcHE2   107:WRKSRSGKVEVPYTIS-RAFPSHERARISRAMDSFHRQSCIRFVPRS-RQRAFITIENRDGCFSSLGRTG 174
MfHE1    78:WRKSSDGIVEVPYVLS-SDFSYYDKLIIDNAMAAFHSTTCIRFVPLS-SQYDYISIENRDGCFSSLGRTG 145
MfHE2    79:WKKSSNGLVEVPYTLS-SDFSYYEKMKLEYALATFHRKTCVRFVPRS-SQNDYVSIENRNGCESSLGRSG 146
ZHE1     76:WKKNANNIVEVPYVVS-GEFSINDKSVIANAISIFHAQTCIRFVPRS-IQADYLSIENKDGCYSAIGRTG 143
ZHE2     76:WKKNSSNFVEVPYIVS-SEYSATEISVIQKAMSGIHNKTCIRFVPRI-SQTDYISIENQDGCFAFIGKKG 143
LoHE1    77:WKKNANNLVEVPYIVS-SEYTSNQISIIQNAMASFHRKTCIRFVPRS-SQIDYISIESKSGCYSSLGKTG 144
LoHE2    78:WKKNANNIVEVPYIVS-SEYTYYEKSTIQSAMTSFASKTCIRFVPRS-SQIDYISIENLDGCYSSLGRTG 145
NeHE1    69:WKKSPTGLVQVPFTVS-ADFSSSDRAVIAGAMATFHSKTCIRFVSRT-IESDYLSIESRDGCYSPVGRSG 136
NeHE2    69:WKKSPTGLVQVPFTVS-ADFSSSDRAVIAGAMATFHSKTCIRSVSRT-IESDYLSIESRDGCYSPVGRSG 136
EeHE1    68:WKKSSNGLVQVPYVVS-TDFSSLDMDVITNAMASFRKKTCIRFVPRT-VEPDYISIKSLDGCYSAVGKTG 135
EeHE2    68:WKKSSNGLVQVPYVVS-TDFSSLDMDVIKNAMASFRKKTCIRFVPRT-VEPDYISIKSRDGCYSAVGKTG 135
CfHE1    68:WKKSSNGLVEVPYTLS-SVFSSSDNTVIANAMATFHSKTCIRFISRT-NQSDYLSIESKDGCYSYVGKIG 135
CfHE2    68:WKKSSNGLVKVPYTLS-SVFSSSDTTAIANAMASFHNKTCIRFVPRT-SETDYIKIESNDGCYSDVGKTG 135
CfHE3    69:WKKSSNGLVKAPYTLS-SDFSSSDTTVVANAMASFHNKTCIRFVPRT-SETDYISIKNNDGCYSDVGKTG 136
Euteleostei clade I
AyHCE    75:WRKSPNGQVMVAYTIS-NEYSPSERYLIEGALRAFASASCVRLVPRT-SFYDYINIVSADGCYSALGRQG 142
SnHCE    77:WRKDPAGHVTVPFTVS-NAFPYHETKKIERALQAIQSKTCIRFVPRQ-NEKDYISVENRGGCYSSLGRVG 144
CdHCE   120:WKKSSNGLVTIPYTVS-RAYTSAERSRIVSAMQSFHRTTCIRFVPRQ-NQKDHISVESRGGCYSSLGRTG 187
FgHCE    75:WPKGSNGLVVIPYTIS-NVYADWERDTIEYAMQSFHSTTCIRFVPRT-NEYDYIMVENGDGCYSSLGKEG 142
TnHCE    74:WPKNSNGLVVIPYTIG-YEYQNWERQAIETALQSFHGSTCIRFTPRK-NEYDYIKVESGDGCYSSLGRVG 141
GaHCE1   78:WKKASNGLVTIPFVIS-NEFTGAEKQVIDRGLKSFHTGTCIRFVPRS-NENDHISIESRGGCFSSMGRTG 145
GaHCE2   78:WKKASNGLVTIPFVIS-NEFTGAETQVIDRGLKSFHTGTCIRFVPRS-NENDHISIESRGGCFSSMGRTG 145
MHCE     82:WKKASNGLVVIPYVIS-SEYSGGEVATIEGAMRAFNGKTCIRFVRRT-NEYDFISVVSKTGCYSELGRKG 149
FHCE     78:WPKASNGKVVIPYVIG-REFSGYERGIIEGGMRAFSGPTCIRFIPRT-NERDFISVVSKQGCWSELGKTG 145
HhHCE1   77:WGKSSNDLVMIPFTVS-SAFTSWERQKINYAMEAFHSWTCLRFVPRQ-NEYDYISIENRVGCFSSLGRTG 144
HhHCE2   78:WRKASNGLVTIPFTMS-SDFTSSERQKIEDAMKTFHSNTCLRFVPRQ-NEYDHISIENRAGCYSYLGRTG 145
PoHCE    75:WKKASNGQVMIPFTVS-SEFTSWERQKIDRAMKAFHSRTCLRFVPRQ-NQYDYISIENKVGCFSSLGRVG 142
MsHCE2   78:WNKASSGYIEVPYTIEDSRFTPSDRKEIEHAVQSFHSKTCIRFVPRG-NQKDYISFESLSGCYSSLGRIG 146
RbHCE2   78:WNKASSGYVEVPYTIEDSRFTPSNRKEIEHAVQSFHSKTCIHFVPRG-NQKDYISFESLNGCYSSLGRIG 146
PkHCE2   71:WRRNSDGQVVVPYILEDNRFSPSDRQKIKSALLSFQDLTCIRFVPRG-NEKDYISYESQRGCFSTLGRAG 139
MsHCE1   79:WNKGPDGLVEVPYTVS-SSFSSSDKQGIENALRAFTSKTCIRFVPWQ-NQVDFISYEPKDGCWSPLGRVG 146
RbHCE1   80:WKKGSNGLVEVPYTVS-SSFSSSDKQGIENALLAFTSKTCIRFVPWQ-NQVDFISYEPRDGCWSSLGRVG 147
PkHCE1   71:WRKGSNGKVEVPYVVS-SEFSSNERQTIQKAVKDINSKTCIHFVPRQ-NQADYLSYEPRDGCWSSLGRQG 138
          // F1 →                                F2 →
```

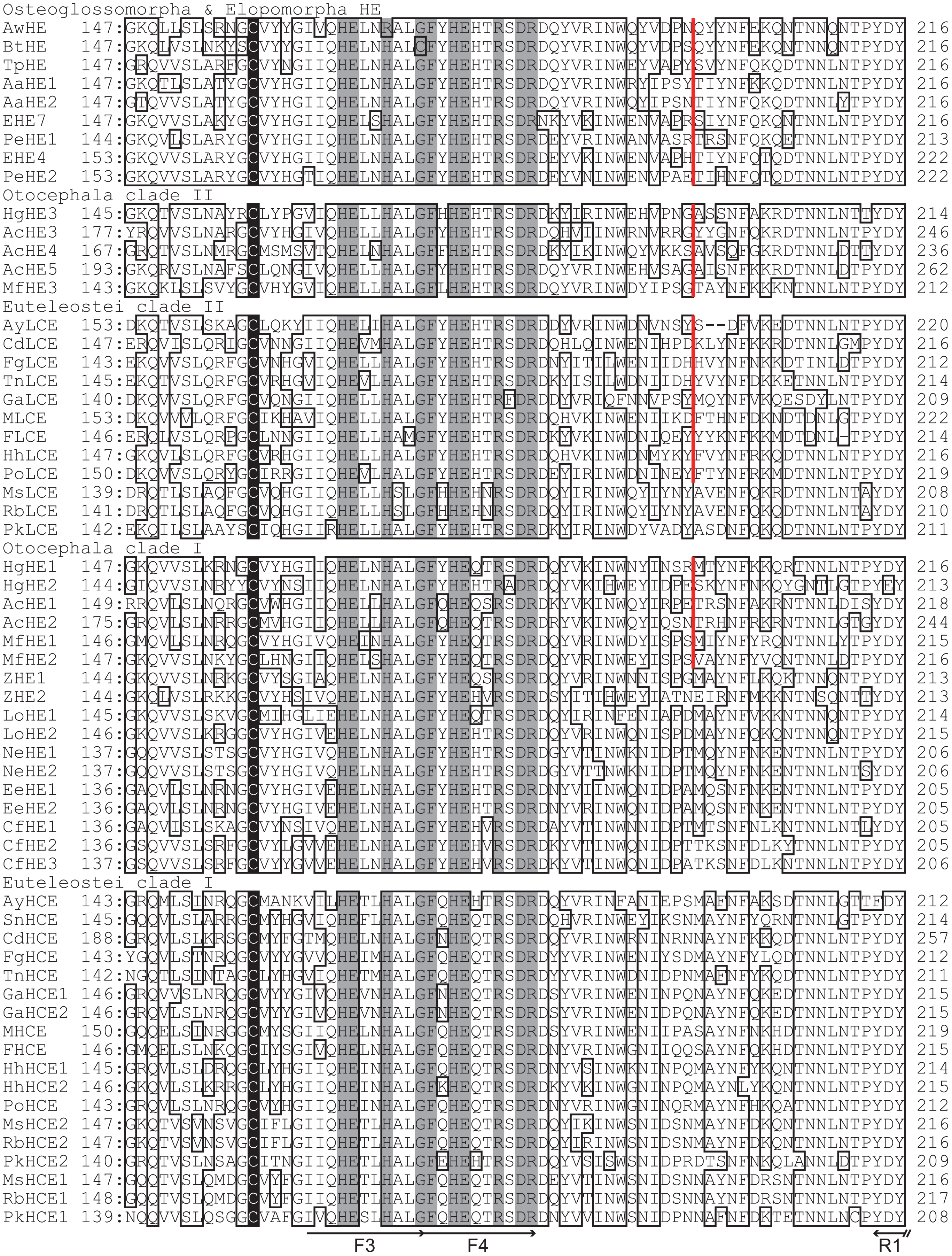
Osteoglossomorpha & Elopomorpha HE
AwHE 147:GKQLLSLSRNGCVYYGIVQHELNRALGFYHEHTRSDRDQYVRINWQYVDPNQYYNFEKQNTNNQNTPYDY 216
BtHE 147:GKQLVSLNKYSCVYYGIIQHELNHALCFYHEHTRSDRDQYVRINWQYVDPSQYYNFEKQNTNNQNTPYDY 216
TpHE 147:GRQVVSLARFGCVYNGIIQHELNHALGFYHEHTRSDRDQYVRINWEYVAPYSVYNFQKQDTNNLNTPYDY 216
AaHE1 147:GKQTLSLATYGCVYHGIIQHELNHALGFYHEHTRSDRDQYVRINWRYIPSYTIYNFKKQDTNNLNTPYDY 216
AaHE2 147:GTQVVSLATYGCVYHGIIQHELNHALGFYHEHTRSDRDQYVRINWQYIPSNTIYNFQKQDTNNLYTPYDY 216
EHE7 147:GKQVVSLAKYGCVYHGIIQHELSHALGFYHEHTRSDRNKYVKINWENVAPRSIYNFQKQNTNNLNTPYDY 216
PeHE1 144:GKQVLSLARYGCVYHGIIQHELNHALGFYHEHTRSDRDEYVRINWANVASRTRSNFQKQETNNLNTPYDY 213
EHE4 153:GKQVVSLARYGCVYHGIIQHELNHALGFYHEHTRSDRDEYVKINWENVAPHTIYNFQTQDTNNLNTPYDY 222
PeHE2 153:GKQVVSLARYGCVYHGTIQHELNHALGFYHEHTRSDRDEYVNINWENVPAETIHNFQTQDTNNLNTPYDY 222
Otocephala clade II
HgHE3 145:GKQTVSLNAYRCLYPGVIQHELLHALGFHHEHTRSDRDKYIRINWEHVPNGASSNFAKRDTNNLNTTYDY 214
AcHE3 177:YRQVVSLNARGCVYHGIVQHELLHALGFYHEHTRSDRDQHVIINWRNVRRGYYGNFQKRDTNNLNTPYDY 246
AcHE4 167:GRQTVSLNMRGCMSMSVTQHELNHALGFFHEHTRSDRDKYIKINWQYVKKSAVSQFGKRDTNNLDTTYDY 236
AcHE5 193:GKQRVSLNAFSCLQNGIVQHELLHALGFYHEHTRSDRDQYVRINWEHVSAGAISNFKKRDTNNLDTPYDY 262
MfHE3 143:GKQKLSLSVYGCVHYGVIQHELLHALGFLHEHTRSDRDQYVRINWDYIPSGTAYNFKKKNTNNLNTPYDY 212
Euteleostei clade II
AyLCE 153:DKQTVSLSKAGCLQKYIIQHELIHALGFYHEHTRSDRDDYVRINWDNVNSYS--DFVKEDTNNLNTPYDY 220
CdLCE 147:ERQVISLQRIGCVNNGIIQHEVMHALGFYHEHTRSDRDQHLQINWENIHPDKLYNFKKRDTNNLGMPYDY 216
FgLCE 143:EKQVVSLQRFGCVNHGVIQHELLHALGFYHEHTRSDRDNYITILWENIIDHHVYNFNKKDTINLDTPYDY 212
TnLCE 145:EKQTVSLQRFGCVRHGVIQHEVLHALGFYHEHTRSDRDKYISILWDNIIDHYVYNFDKKETNNLNTPYDY 214
GaLCE 140:DKQVVSLQRFGCVQNGIIQHELLHALGFYHEHTRFDRDDYVRIQFNNVPSYMQYNFVKQESDYLNTPYDY 209
MLCE 153:DKQVVVLQRFGCIKHAVIQHELLHALGFYHEHTRSDRDQHVKINWENIIKDFTHNFDKNDTDNLGTPYDY 222
FLCE 146:ERQLVSLQRPGCLNNGIIQHELLHAMGFYHEHTRSDRDKYVKINWDNIQEYYYKNFKKMDTDNL-TPYDY 214
HhLCE 147:GKQVLSLQRFGCVRHGIIQHELLHALGFYHEHTRSDRDQHVKINWDNMYKYFVYNFRKQDTNNLNTPYDY 216
PoLCE 150:DKQVVSLQRYGCIRRGIIQHEVLHALGFYHEHTRSDRDEYIRINWDNINEYFTYNFRKMDTNNLNTPYDY 219
MsLCE 139:DRQTLSLAQFGCVQHGIIQHELLHSLGFHHEHNRSDRDQYIRINWQYIYNYAVENFQKRDTNNLNTAYDY 208
RbLCE 141:DRQTLSLAQFGCVQHGIIQHELLHSLGFHHEHNRSDRDQYIRINWQYIYNYAVENFQKQDTNNLNTAYDY 210
PkLCE 142:EKQILSLAAYSCIQHGIIRHELLHALGFYHEHTRSDRDKYIRINWDYVADYASDNFQKQDTNNLNTPYDY 211
Otocephala clade I
HgHE1 147:GKQVVSLKRNGCVYHGIIQHELNHALGFYHEQTRSDRDQYVKINWNYINSRMTYNFKKQRTNNLNTPYDY 216
HgHE2 144:GIQVVSLNRYYCVYNSIIQHELNHALGFYHEHTRADRDQYVKIHWEYIDPESKYNFNKQYGNTLGTPYEY 213
AcHE1 149:RRQVLSLNQRGCVWHGIIQHELLHALGFQHEQSRSDRDKYVKINWQYIRPETRSNFAKRNTNNLDISYDY 218
AcHE2 175:GRQVLSLNRRGCMVHGIIQHELLHALGFQHEQTRSDRDRYVKINWQYIQSNTRHNFRKRNTNNLGTGYDY 244
MfHE1 146:GMQVLSLNRQGCVYHGIVQHEINHALGFYHEQTRSDRDQYVRINWDYISPSMIYNFYRQNTNNLYTPYDY 215
MfHE2 147:GKQVVSLNKYGCLHNGIIQHELSHALGFYHEQTRSDRDQYVRINWEYISPSVAYNFYVQNTNNLDTPYDY 216
ZHE1 144:GKQVVSLNRKGCVYSGIAQHELNHALGFYHEQSRSDRDQYVRINWNNISPGMAYNFLKQKTNNQNTPYDY 213
ZHE2 144:GKQLVSLRKKGCVYHSIVQHELNHALGFYHEHVRSDRDSYITIHWEYIATNEIRNFMKKNTNSQNTTYDY 213
LoHE1 145:GKQVVSLSKVGCMIHGLIEHELNHALGFYHEQTRSDRDQYIRINFENIAPDMAYNFVKKNTNNQNTPYDY 214
LoHE2 146:GKQVVSLKRGGCVYHGIVEHELNHALGFYHEHTRSDRDQYVRINWQNISPDMAYNFQKQNTNNQNTPYDY 215
NeHE1 137:GQQVVSLSTSGCVYHGIVQHELNHALGFYHEHTRSDRDGYVTINWKNIDPTMQYNFNKENTNNLNTPYDY 206
NeHE2 137:GQQVVSLSTSGCVYHGIVQHELNHALGFYHEHTRSDRDGYVTTNWKNIDPTMQYNFNKENTNNLNTSYDY 206
EeHE1 136:GAQVLSLNRNGCVYHGIVEHELNHALGFYHEHTRSDRDNYVIINWQNIDPAMQSNFNKENTNNLNTPYDY 205
EeHE2 136:GAQVLSLNRNGCVYHGIVEHELNHALGFYHEHTRSDRDNYVRINWQNIDPAMQSNFNKENTNNLNTPYDY 205
CfHE1 136:GAQVISLSKAGCVYNSIVQHELNHALGFYHEHVRSDRDAYVTINWNNIDPTMTSNFNLKNTNNLNTLYDY 205
CfHE2 136:GSQVVSLSRFGCVYLGVVEHELNHALGFYHEHVRSDRDKYVTINWQNIDPTTKSNFDLKYTNNLNTPYDY 205
CfHE3 137:GSQVVSLSRFGCVYYGVVEHELNHALGFYHEHVRSDRDKYVTINWQNIDPATKSNFDLKNTNNLNTPYDY 206
Euteleostei clade I
AyHCE 143:GRQMLSINRQGCMANKVILHETLHALGFQHEHTRSDRDQYVRINFANIEPSMAFNFAKSDTNNLGTTFDY 212
SnHCE 145:GQQVLSLARRGCMYHGVIQHEFLHALGFQHEQTRSDRDQHVRINWEYIKSNMAYNFYQRNTNNLGTPYDY 214
CdHCE 188:GRQVLSLKRSGCMYFGTMQHELNHALGFNHEQTRSDRDQYVRINWRNINRNNAYNFKKQDTNNLNTPYDY 257
FgHCE 143:YGQVLSTNRQGCVYYGVVQHEIMHALGFQHEQTRSDRDYYVRINWENIYSDMAYNFYLQDTNNLNTPYDY 212
TnHCE 142:NGQTLSINTAGCLYHGVIQHETMHALGFQHEQTRSDRDYYVRINWDNIDPNMAFNFYKQDTNNLNTPYDY 211
GaHCE1 146:GRQVVSLNRQGCVYYGIVQHEVNHALGFNHEQTRSDRDSYVRINWENINPQNAYNFQKEDTNNLNTPYDY 215
GaHCE2 146:GRQVLSLNRQGCVYYGIVQHEVNHALGFNHEQTRSDRDSYVRINWENIDPQNAYNFQKEDTNNLNTPYDY 215
MHCE 150:GQQELSINRGGCMYSGIIQHELNHALGFQHEQTRSDRDSYVRINWENIIPASAYNFNKHDTNNLNTPYDY 219
FHCE 146:GMQELSLNKQGCIYSGIVQHELNHALGFQHEQTRSDRDNYVRINWGNIIQQSAYNFQKHDTNNLNTPYDY 215
HhHCE1 145:GRQVLSLDRQGCLYHGIIQHEINHALGFQHEQTRSDRDNYVSINWKNINPQMAYNFYKQNTNNLNTPYDY 214
HhHCE2 146:GKQVLSLKRRGCLYHGIIQHEINHALGFKHEQTRSDRDKYVKINWGNINPQMAYNLYKQNTNNLNTPYDY 215
PoHCE 143:GRQVLSLNRQGCVYHGIIQHEINHALGFQHEQTRSDRDNYVRINWGNINQRMAYNFHKQATNNLNTPYDY 212
MsHCE2 147:GKQTVSVNSVGCIFLGIIQHETLHALGFQHEQTRSDRDQYIKINWSNIDSNMAYNFDKQNTNNLNTPYDY 216
RbHCE2 147:GKQTVSVNSVGCIFLGIIQHETLHALGFQHEQTRSDRDQYIRINWSNIDSNMAYNFDKQNTNNLNTPYDY 216
PkHCE2 140:GRQTVSLNSAGCITNGIIQHETLHALGFEHEHTRSDRDQYVSISWSNIDPRDTSNFNKQLANNLDTPYDY 209
MsHCE1 147:GQQTVSLQMDGCVYFGIIQHETLHALGFQHEQTRSDRDEYVTINWSNIDSNNAYNFDRSNTNNLNTPYDY 216
RbHCE1 148:GQQTVSLQMDGCVYFGIIQHETLHALGFQHEQTRSDRDQYVTINWSNIDSNNAYNFDRSNTNNLNTPYDY 217
PkHCE1 139:NQQVVSLQSGGCVAFGIVQHESLHALGFQHEQTRSDRDNYVVINWSNIDPNNAFNFDKTETNNLNCPYDY 208
F3
F4
R1

```
Osteoglossomorpha & Elopomorpha HE
AwHE   217:GSIMHYGRTAFTIQYGMDTITPIPNPNVPIGQRNGLSQIDILRINKLYGCX------------------- 267
BtHE   217:GSVMHYGRTAFTIQYGMETITPIPDPTVPIGQMNGLSQMDVLRINKLYGC*------------------- 266
TpHE   217:SSIMHYGRSAFSTQYGQETITPIPDPNQPIGQRRNLSDLDIQRINRLYGCX------------------- 267
AaHE1  217:TSVMHYGRTAFSNRKGRETITPIPNANQPIGQRTSLSIMDIQRISKLYSCX------------------- 267
AaHE2  217:TSVMHYGRTAFPNRKGRETITPIPNANQPIGQRTSLSIMDIQRINKLYSCX------------------- 267
EHE7   217:TSIMHYGKAAFSTN-GKDTITPIPNPKQSIGQRRSMSKGDILRIKKLYNCCKKRNI*-------------- 271
PeHE1  214:TSIMHYGKTAFSTN-GKDTITPIPNPNQSIGQRRSLSRGDILRIKKLYSCNNDX----------------- 266
EHE4   223:TSIMHYGRTAFSTN-GMDTITPVPNPNQSIGQRRSMSRGDILRIKKLYSC*------------------- 271
PeHE2  223:TSIMHYGSTAFSAN-GLDTITPVPKPNQPIGQGTSLSRGDILRINKLYSCX------------------- 272
Otocephala clade II
HgHE3  215:SSLMHYGKYSFTSSFGKATIIPIPDPEVTIGQRTDLSETDIFELNKIYKCDEQ*----------------- 267
AcHE3  247:GSVMHYGRYEFSNRSG-PTITPRRNPGFTLGLQQAMTEIDILKVNRLYECDVPK*---------------- 299
AcHE4  237:NSIMHYDRFAFTNTPGRETLTPIPKR-VSVGPRARLSKTDIERINKLY-CGATGPTTTTTTIPPPTTTKT 304
AcHE5  263:SSVMHYGRYADSTSRLHPTITPIPDASMEIGQRNELSEIDILKINKLYKCGGLQ*---------------- 316
MfHE3  213:GSIMHYGRTAFSTEYGKDTITPIPDSSVEIGQRQEMSDIDIQRINELYECDV*------------------ 264
Euteleostei clade II
AyLCE  221:SSIMHYEKYAFARDKSMPSITPIPDPNVVLGQRVGMSAIDIKRINILYNC*------------------- 270
CdLCE  217:QSVTHYGRRAFSTV-WKDTITPIPDASVRIGKSNGLSGIDIQKINKLYKC*------------------- 265
FgLCE  213:SFIMHYGRGAFGMN-RKETLIPYPDSSVEIGQRDGMSEIDVLRVNRLYKCHGY*---------------- 264
TnLCE  215:GSIMHYGRDAFGIN-RKETMIPIPDSSVDIGQREVMSAIDVLRVNKLYKCDGY*---------------- 266
GaLCE  210:SSVMHYGKTAFA-DAGTESIIPIPDSSVPIGQRVTMSDIDILRIKRLYKC*------------------- 258
MLCE   223:GSIMHYGRTAFGKD-RKETITPIPNPKAAIGQTERMSDIDILRVNKLYKC*------------------- 271
FLCE   215:SSVMQYGKTAFGKN-RAESITPIPDPNVPIGQREGMSDTDILRVNKLYKCWSYIG*-------------- 268
HhLCE  217:SSIMHYGRTAFGKH-RSETIIPIPDSSVPIGQRDELSKTDILRINRLYKCGNY*---------------- 268
PoLCE  220:SSVMHYGRTAFGRF-RAETMTPIPDPSVAMGQRNGMSHIDVLRINRLYKCWSYIG*-------------- 273
MsLCE  209:SSVMHYDRTAYTNDYGKETITPIPDPSVAIGQRQGMSDIDVLRVNKLYQC*------------------- 258
RbLCE  211:SSVMHYDRTAYTNNYGKETITPIPDPSVAIGQRQGMSDIDVLRVNKLYQC*------------------- 260
PkLCE  212:SSVMHYDKTAFSTDLAKETITPIPDESVRIGQRKEMSDIDILRINKLYKCQ*------------------ 262
Otocephala clade I
HgHE1  217:SSVMHYGRTAFTVQNGKETITPIPNPNVRIGQRQGMSTTDILRINKLYGC*------------------- 266
HgHE2  214:SSVMHYGRTAFSNQNGKSAITTIPDPTVSIGQAQGLSTTDILRINKLYGC*------------------- 263
AcHE1  219:GSVMHYGRYAFTTKRGVETITPIPDSSVQIGQRRGMSKSDIQRINKLYQCCSKE*---------------- 272
AcHE2  245:SSVLHYGRYAFTTRRGAETITPYPNRRVQIGQRTRMSRNDILRLNRLYRC*------------------- 294
MfHE1  216:SSIMHYGRTAFAVQYGVETITPIPDASVQIGQRQGLSDIDIQRINLLYGC*------------------- 265
MfHE2  217:SSVMHYGRTAFSNQYGKETITPIPDESVQIGQSQGLSDIDILRINKLYDCNM*------------------ 268
ZHE1   214:GSLMHYGKTAFAIQPGLETITPIPDENVQIGQRQGLSKIDILGINKLYGC*------------------- 263
ZHE2   214:GSIMHYGKTAFTTVKGKETMTPYPDETVPIGKAKEMSDIDILRINMMYSCNISDDLKI*------------ 271
LoHE1  215:GSIMHYRRTAFAIQAGLVTITPIPDESVSIGQRKEMSDIDILRINKLYGC*------------------- 264
LoHE2  216:GSVMHYGNTAFATQYGLETITPIPDASVQIGQRQELSNIDILRINKLYGC*------------------- 265
NeHE1  207:SSVMHYGKTAFTIN-GLDTITPIPNASVQIGQRVDLSTIDILRINTLYKC*------------------- 255
NeHE2  207:SSVMHYGKTAFTIN-GLDTITPIPNVSVQIGQRVDLSTIDILRINTLYKC*------------------- 255
EeHE1  206:SSVMHYGNTAFSIN-GMDTITPIPDEWVAIGQRVELSTIDVKRIKILYEC*------------------- 254
EeHE2  206:SSVMHYGNTAFSIN-GMDTITPIPDEWVAIGQRVELSTIDVKRIKILYEC*------------------- 254
CfHE1  206:SSVMHYGRTAFSIN-GLDTITPIPDPSVKIGQSTELSATDILRIKTLYNC*------------------- 254
CfHE2  206:SSVMHYGNTAFSIN-DLDTITPIPDPSVMIGQRQELSTIDIKRINILYNC*------------------- 254
CfHE3  207:SSVMHYGKTAFSIN-GLDTITPTPDPSVMIGQRQELSTIDIKRINVLYKC*------------------- 255
Euteleostei clade I
AyHCE  213:GSIMQYPRQAFAINPSIDTIIPIPNPYVDMGVATTFSRIDIQRLNTLYRC*------------------- 262
SnHCE  215:SSIMHYGRTAFSTRYGKETITPIPNPNVKIGQRRGLSRIDVLRINRLYEC*------------------- 264
CdHCE  258:SSIMHYGRTAFSIN-GRDSITPIPNSRVQLGQRKGMTRNDILRINRLYRC*------------------- 306
FgHCE  213:SSIMHYGRTAFSMYNGVETITPIPDPYVQIGQREGMSYWDIQRVNLLYGC*------------------- 262
TnHCE  212:GSVMHYGRTAFTMYAGVDTITPIPDSRVQIGQRNGLSYWDIQRVNLLYGC*------------------- 261
GaHCE1 216:SSIMHYGKTAFSIN-GRDSITPIPNENAQIGQRNGMSDWDIKRINLLYGC*------------------- 264
GaHCE2 216:SSVMHYGNTAFSIN-GRDSITPIPNANAQIGQRNGMSYWDIKRINLLYGC*------------------- 264
MHCE   220:SSIMHYGRDAFSIAYGRDSITPIPNPNVPIGQRNGMSRWDITRINVLYNCR*------------------ 270
FHCE   216:SSIMHYGRDAFAIAYGRETITPFPNPNVPIGQRQGLSRWDVQRINMLHGC*------------------- 265
HhHCE1 215:SSIMHYGRTAFSIQYGKDSITPIPDPNVQIGQRRGMSYWDIVRINALYDC*------------------- 264
HhHCE2 216:SSIMHYGRTAFSIKYGQDSITPIPDPNVEIGQRKGLSSWDIKRINLLYRC*------------------- 265
PoHCE  213:SSIMHYGKTAFSIQYGRDSITPIPNPNVQIGQRQGMSYWDIMRINRLYGC*------------------- 262
MsHCE2 217:SSVMHYGKTAFSIN-GMDTITPIPNPDVSIGQRQGMSTTDILRINRLYSC*------------------- 265
RbHCE2 217:SSVMHYGKTAFSTN-GMDTITPIPNPDVSIGQRQGLSTTDILRINRLYGC*------------------- 265
PkHCE2 210:SSVMHYGKMAFSIN-GQETITTIPDHNMPIGQTVQMSDIDILTINKLYNCSQ*----------------- 260
MsHCE1 217:SSVMHYGKTAFSIN-GMDTITPIPNPDVPIGQRQGLSTTDILRINRLYGC*------------------- 265
RbHCE1 218:SSVMHYGKTAFSVN-GMDTITPIPNPNVPIGQRQGLSTTDILRINRLYGC*------------------- 266
PkHCE1 209:GSVMHYDNTAFSIN-GEDTITPIPNPNVPIGQRVGLSATDILKINRLYNC*------------------- 257
```

R1 R2

```
Osteoglossomorpha & Elopomorpha HE
AwHE   267:---------------------------                                            267
BtHE   266:---------------------------                                            266
TpHE   267:---------------------------                                            267
AaHE1  267:---------------------------                                            267
AaHE2  267:---------------------------                                            267
EHE7   271:---------------------------                                            271
PeHE1  266:---------------------------                                            266
EHE4   271:---------------------------                                            271
PeHE2  272:---------------------------                                            272
Otocephala clade II
HgHE3  267:---------------------------                                            267
AcHE3  299:---------------------------                                            299
AcHE4  305:TTTTAPTTTTTEAPVTKTPCPYDDGE*                                            330
AcHE5  316:---------------------------                                            316
MfHE3  264:---------------------------                                            264
Euteleostei clade II
AyLCE  270:---------------------------                                            270
CdLCE  265:---------------------------                                            265
FgLCE  264:---------------------------                                            264
TnLCE  266:---------------------------                                            266
GaLCE  258:---------------------------                                            258
MLCE   271:---------------------------                                            271
FLCE   268:---------------------------                                            268
HhLCE  268:---------------------------                                            268
PoLCE  273:---------------------------                                            273
MsLCE  258:---------------------------                                            258
RbLCE  260:---------------------------                                            260
PkLCE  262:---------------------------                                            262
Otocephala clade I
HgHE1  266:---------------------------                                            266
HgHE2  263:---------------------------                                            263
AcHE1  272:---------------------------                                            272
AcHE2  294:---------------------------                                            294
MfHE1  265:---------------------------                                            265
MfHE2  268:---------------------------                                            268
ZHE1   263:---------------------------                                            263
ZHE2   271:---------------------------                                            271
LoHE1  264:---------------------------                                            264
LoHE2  265:---------------------------                                            265
NeHE1  255:---------------------------                                            255
NeHE2  255:---------------------------                                            255
EeHE1  254:---------------------------                                            254
EeHE2  254:---------------------------                                            254
CfHE1  254:---------------------------                                            254
CfHE2  254:---------------------------                                            254
CfHE3  255:---------------------------                                            255
Euteleostei clade I
AyHCE  262:---------------------------                                            262
SnHCE  264:---------------------------                                            264
CdHCE  306:---------------------------                                            306
FgHCE  262:---------------------------                                            262
TnHCE  261:---------------------------                                            261
GaHCE1 264:---------------------------                                            264
GaHCE2 264:---------------------------                                            264
MHCE   270:---------------------------                                            270
FHCE   265:---------------------------                                            265
HhHCE1 264:---------------------------                                            264
HhHCE2 265:---------------------------                                            265
PoHCE  262:---------------------------                                            262
MsHCE2 265:---------------------------                                            265
RbHCE2 265:---------------------------                                            265
PkHCE2 260:---------------------------                                            260
MsHCE1 265:---------------------------                                            265
RbHCE1 266:---------------------------                                            266
PkHCE1 257:---------------------------                                            257
```
